# Autologous Bone-Marrow-Derived-Mononuclear-Cells-Enriched Fat Transplantation in Breast Augmentation: Evaluation of Clinical Outcomes and Aesthetic Results in a 30-Year-Old Female

**DOI:** 10.1155/2013/782069

**Published:** 2013-08-19

**Authors:** Dmitry Bulgin, Erik Vrabic, Enes Hodzic

**Affiliations:** ^1^Polyclinic “ME-DENT,” 18 Istarska, 52210 Rovinj, Croatia; ^2^Department of Plastic and Reconstructive Surgery, University Clinical Centre (UCC), 5 Ljubljanska, 2000 Maribor, Slovenia

## Abstract

Autologous fat transfer (lipofilling) is becoming an invaluable tool for breast augmentation as well as for breast reconstruction. Autologous lipofilling has several advantages, including biocompatibility, versatility, natural appearance, and low donor site morbidity. The main limitation is unpredictable fat graft resorption, which ranges from 25% to 80%, probably as a result of ischaemia and lack of neoangiogenesis. To obviate these disadvantages, several studies have searched for new ways of increasing the viability of the transplanted fat tissue. One promising approach is to enrich the fat graft with autologous bone-marrow-derived mononuclear cells (BMMNCs) before transplantation. BMMNCs produce many angiogenic and antiapoptotic growth factors, and their secretion is significantly enhanced by hypoxia. All of these mechanisms of actions could be beneficial for the stimulation of angiogenesis in ischemic tissues by BMMNCs administration. In our aesthetic surgery practice, we use fat transplantation enriched with BMMNCs, which caused a significant improvement in survival of fat grafts, compared with that of traditional lipofilling. Our experience with freshly isolated autologous fat enriched with BMMNCs for breast augmentation procedures is presented. The concept of this surgical and tissue handling technique is based on ability of BMMNCs to stimulate blood vessel growth.

## 1. Introduction

After numerous experiments since 1893 [1, 2], autologous fat transplantation has become a well-established and frequently applied method of soft tissue augmentation for both cosmetic and reconstructive purposes. Tissue augmentation by fat grafting does have several advantages, and many promising results of autologous fat grafts have been published recently [3–8]. Autologous fat is an excellent and extremely promising soft-tissue filler, given its abundance and ease of harvest [9–13]. Selecting suitable indications and correct surgical techniques, low complication rate, and positive results make autologous fat grafting an ideal method for breast augmentation [14, 15].

Published clinical experience in over 2,000 patients who received autologous fat grafts showed no evidence of increased risk of development, metastasis, or recurrence of breast cancer [16–19]. The most significant drawback to autologous fat grafting remains its largely unpredictable rate of resorption, low rate of graft survival due to partial necrosis, and scattered microcalcifications, followed by dispersed radiolucent oil cysts [20–22]. Numerous *in vitro* and *in vivo* studies on fat graft viability have recently been done. According to results of these studies, the use of bone-marrow-derived cells is a novel approach to survival of fat grafts [23–25].

The autologous application of BMMNCs which are not expanded *ex vivo* has medicolegal advantages for clinical use [26]. BMMNCs as a huge source of bone-marrow-derived mesenchymal stem cells (BMMSCs) [27–31] represent a potential key component in the field of regenerative medicine and tissue engineering [32–34]. BMMSCs are multipotent and secrete many kinds of growth factors to regenerate tissues. During the past decade, numerous studies have provided preclinical and clinical data on the safety and efficacy of BMMSCs, supporting the use of these cells in a wide range of clinical applications such as plastic surgery, cardiac surgery, orthopedic surgery, and oral and maxillofacial surgery [35–40]. BMMNCs stimulated angiogenesis as well as maturation of the newly formed blood vessels *in vivo*. These mechanisms of actions could be beneficial for the stimulation of angiogenesis in ischemic tissues by BMMNCs administration [41]. According to these characteristics, we suggest that the BMMNCs-enriched lipografts can produce aesthetically acceptable results without the need for repeating treatment sessions, which are necessary with autologous fat transplantation.

## 2. Materials and Methods

### 2.1. Patient

The patient is a 30-year-old female who requested bilateral breast augmentation. The patient signed a detailed informed consent form of the procedure and possible complications.

The patient has been carefully monitored with preoperative and serial postoperative ultrasonograms (preoperatively, at day 1; postoperatively, after 2 weeks, 4 weeks, 3 months, 6 months, and 12 months).

### 2.2. Bone-Marrow-Derived Mononuclear Cells Preparation

**Figure 1:**
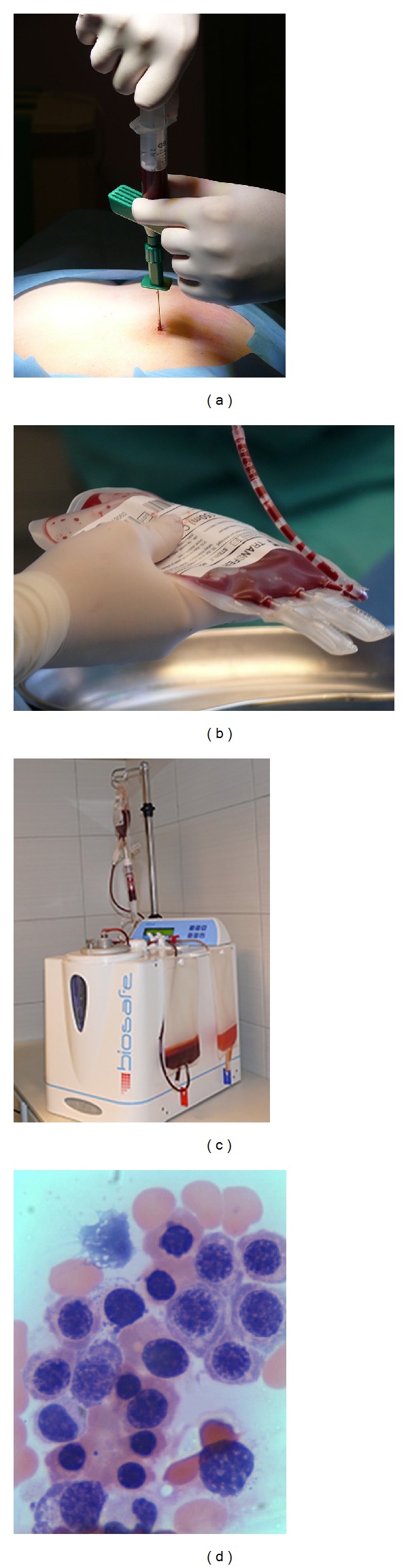
Bone-marrow-derived mononuclear cells preparation: (a) the bone marrow harvesting from posterior iliac crest, (b) collection of bone marrow in plastic bag, (c) the bone marrow processing by using Cell Separation System SEPAX S-100, and (d) qualitative assessment of BMMNCs population by haematoxylin and eosin cytological staining (×400).

Autologous bone marrow from the patient was used as a source for BMMNCs. Under general anesthesia, 70.0 mL of the bone marrow was harvested from posterior iliac crest. BMMNCs were separated according to generic volume reduction protocol by using Cell Separation System SEPAX S-100/a table top centrifuge system (Biosafe Group SA, Eysins, Switzerland). The SEPAX S-100 is a cell processing system that uses a rotating syringe technology that provides both separation through rotation of the syringe chamber (centrifugation) and component transfer through displacement of the syringe piston. The SEPAX S-100 system allows the automated processing of cell components in a functionally closed and sterile environment. The SEPAX S-100 provides centrifugal and axial displacement drive to the chamber on the single-use separation kit, as well as drive to the directional valves. The cell separation process is permanently monitored by an optical sensor, fully automated, and completed within 15 to 20 minutes and required a minimum of (nonspecialized) operator intervention. The generic volume reduction protocol uses a single sedimentation step with centrifugal force of 960 ×g and concentrates the final cell product. After processing of the bone marrow, the final BMMNCs product was suspended in 15 mL autologous plasma. Quantitative assessment of cell population by Sysmex KX-21N cell counter (Sysmex Corp., Kobe, Japan), Trypan Blue exclusion test of cell viability, and *in vitro* haematoxylin and eosin cytological staining was performed to confirm viability and composition of BMMNCs (Figure 1).

### 2.3. Purified Fat and BMMNCs Mixture Preparation and Breast Augmentation Procedure

**Figure 2:**
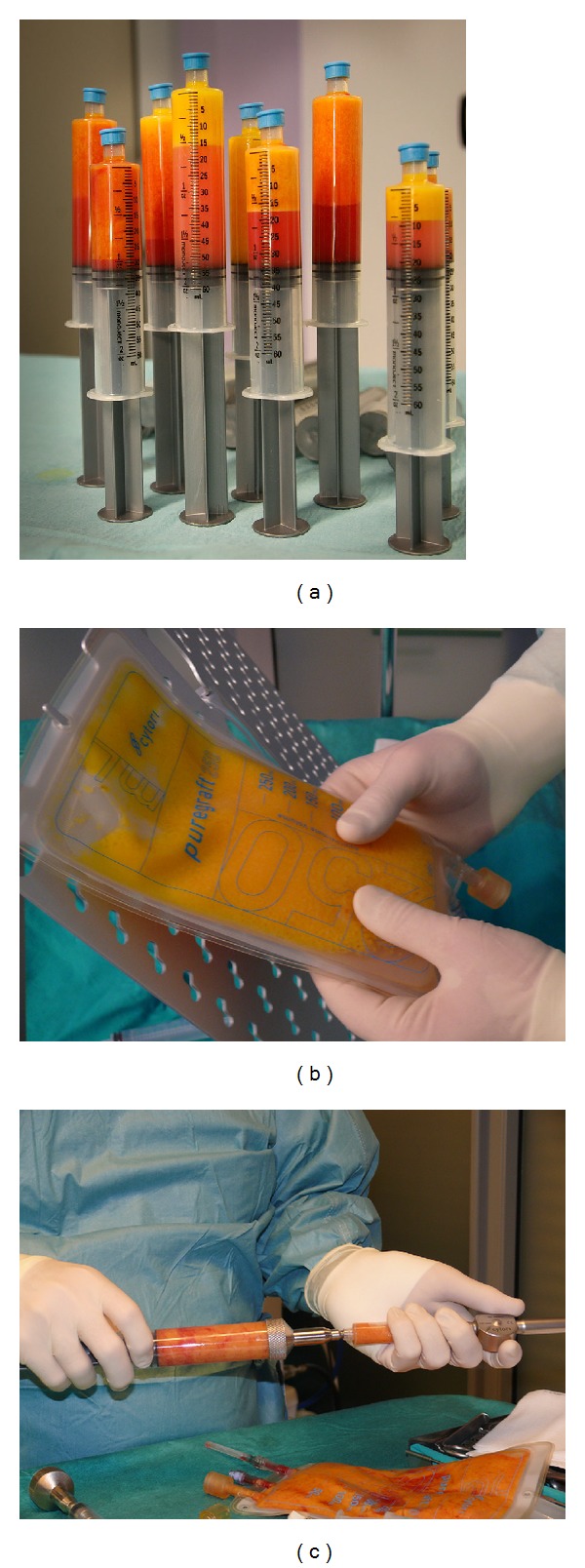
Purified fat and BMMNCs mixture preparation: (a) the harvested fat after tumescent syringe liposuction, (b) the purified fat in Cytori PureGraft 250/PURE System, and (c) the BMMNCs and purified fat mixture transferring to 10 mL syringe.

After BMMNCs preparation during the same anesthetic event, the patient was admitted for the next surgical procedures which included tumescent syringe liposuction [42] by the Coleman technique, which is based on manual aspiration [43, 44]. Fat was harvested mainly from the abdomen, thighs, and flanks. The harvested fat was transferred into a rigorously closed system Cytori PureGraft 250/PURE System (Cytori Therapeutics, Inc., San Diego, CA, USA). The PureGraft 250 System is indicated for autologous fat transfers. The PureGraft System allows the user to prepare fat grafts within the sterile field in less than 15 minutes. PureGraft selectively washes the graft, drains the tumescent fluid, and removes free lipid and debris. After processing the obtained purified fat and 15.0 mL of BMMNCs product were mixed in the same closed sytem (PureGraft 250 System). The BMMNCs and purified fat mixture was transferred to 10 mL syringes for injection directly into the breast by using micro droplet injection device Celbrush (Cytori Therapeutics, Inc., San Diego, USA) (Figure 2).

**Figure 3:**
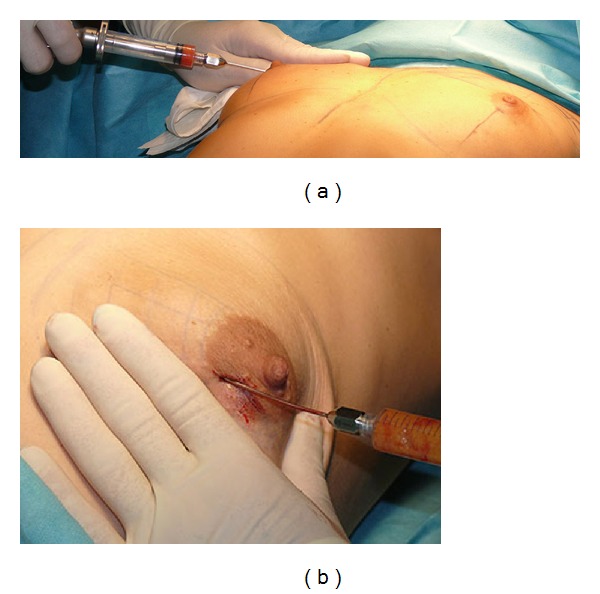
Purified fat and BMMNCs mixture reimplantation: (a) and (b) breast augmentation procedure (bicompartmental grafting).

Cytori's Celbrush is a stainless steel device intended for use in the delivery of an autologous fat graft. The thumb-brush design gives the Celbrush a mechanical advantage that minimizes the buildup of pressure and provides superior tactile feedback during tissue dispersion. The 10 mL Celbrush is designed to deliver approximately 0.50 mL of tissue for each full brush of the operator's thumb. Minimally manipulated fat combined with BMMNCs was reimplanted strictly in two planes only: into the retroglandular and prefascial space and into the superficial subcutaneous plane of the upper pole of the breast (bicompartmental grafting) (Figure 3).

Any intraparenchymal placement was carefully avoided. Total grafted fat volume was 185.0 mL per breast. Average operation time was 3.5 h. Postoperative follow-up was uneventful and no complications were observed.

## 3. Results

**Figure 4:**
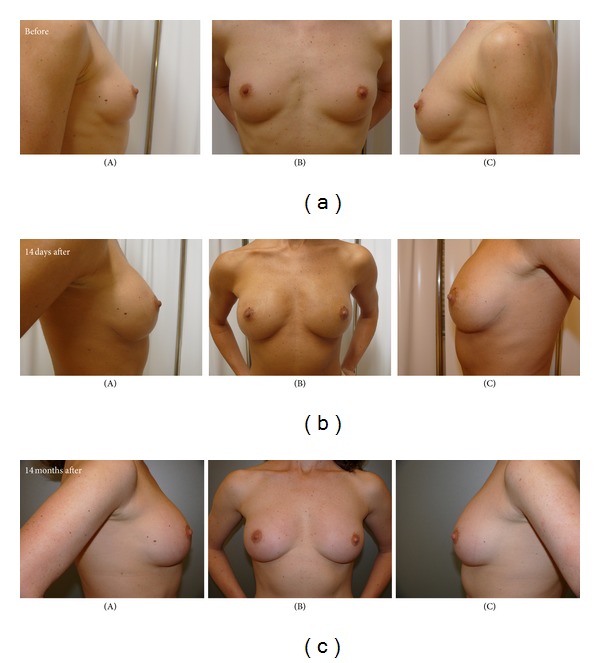
Clinical outcomes and aesthetic results after BMMNCs-enriched lipograft for primary bilateral breast augmentation: preoperative views (top), and postoperative views at 14 days (middle), postoperative views at 12 months (bottom); (A) right view, (B) front view, and (C) left view.

The patient was satisfied with the soft and natural-appearing augmentation. The breast mounds were soft with no subcutaneous induration and visible injection scars. Postoperative atrophy of injected fat was minimal and did not change substantially after 12 months (Figure 4).

The patient demonstrated improvement in circumferential breast measurement (BRM) from baseline state, and breast measurements were stable by 3 months after surgery. The BRM 12 months after surgery had increased 5.5 cm from preoperative measurements. Breast ultrasound showed no evidence of cyst formation or microcalcification.

## 4. Discussion

During the past decade, numerous studies have provided preclinical data on the safety and efficacy of BMMNCs, supporting the use of these cells in future clinical applications. Various clinical outcomes have shown the regenerative capability of BMMNCs in subspecialties of medical fields such as plastic surgery, orthopedic surgery, oral and maxillofacial surgery, and cardiac surgery [35, 39, 40, 45]. These preliminary results suggest that BMMNCs-assisted lipotransfer could be effective and safe for soft tissue augmentation and superior to conventional lipoinjection. BMMNCs are an alternative cell source for obtaining mesenchymal stem cells (MSCs). The idea of using BMMNCs autografts is based on the assumption that MSCs among the mononuclear cells, which are present in only relatively small numbers in bone marrow aspirates, can be easily separated *ex vivo* from the rest of the harvested cells, concentrated in small volume, and immediately implanted into the patient's injured tissue, where the microenvironment will trigger their replication (i.e., cloning) and differentiation into specialized cells. MSCs produce many angiogenic and antiapoptotic growth factors, and their secretion is significantly enhanced by hypoxia [46]. MSCs enhance blood vessel growth not only by production of paracrine-acting factors but also by promoting the endothelial cells differentiation [47]. All of these mechanisms of actions could be beneficial for the stimulation of angiogenesis in transplanted fat by BMMNCs administration. Although an optimal method of cells-assisted autologous fat grafting for primary breast augmentation should be standardized, further strong-evidence-based studies are necessary to confirm the findings of this approach [48, 49].

Breast ultrasound, an accurate and simple imaging technique, plays an important role in follow-up for temporal changes of fat nodules after autologous fat injection. Furthermore, breast ultrasound may avoid unnecessary biopsies [50].

## 5. Conclusions

It was observed that autologous fat combined with freshly isolated BMMNCs possessed excellent handling characteristics, with no adverse tissue reaction and infection. BMMNCs appear to be an ideal population of stem cells for practical regenerative medicine, given that they are plentiful, of autologous tissue origin and thus nonimmunogenic, and more easily available because of minimal ethical considerations.

The advantages of this method for clinical use in primary breast augmentation are as follows:

- the cells do not need to be expanded *in vitro*; they preserve their angiogenic potential to form new blood vessels and promote the proper graft survival;
- the aspirated fat is used as a living scaffold.

Our clinical outcomes showed that the procedure is safe and effective, providing improvement after a single treatment. Further long-term studies are necessary to confirm the favorable results seen in this study.
